# Ultra-Deep Sequencing Characterization of HCV Samples with Equivocal Typing Results Determined with a Commercial Assay

**DOI:** 10.3390/ijms17101679

**Published:** 2016-10-07

**Authors:** Claudia Minosse, Emanuela Giombini, Barbara Bartolini, Maria R. Capobianchi, Anna R. Garbuglia

**Affiliations:** Laboratory of Virology, National Institute for Infectious Diseases “Lazzaro Spallanzani”—IRCCS, Via Portuense 292, Rome 00149, Italy; claudia.minosse@inmi.it (C.M.); emanuela.giombini@inmi.it (E.G.); barbara.bartolini@inmi.it (B.B.); maria.capobianchi@inmi.it (M.R.C.)

**Keywords:** HCV genotyping, HCV subtype, ultra-deep pyrosequencing, mixed HCV infection, indeterminate genotype

## Abstract

Hepatitis C virus (HCV) is classified into seven phylogenetically distinct genotypes, which are further subdivided into related subtypes. Accurate assignment of genotype/subtype is mandatory in the era of directly acting antivirals. Several molecular methods are available for HCV genotyping; however, a relevant number of samples with indeterminate, mixed, or unspecified subtype results, or even with misclassified genotypes, may occur. Using NS5B direct (DS) and ultra-deep pyrosequencing (UDPS), we have tested 43 samples, which resulted in genotype 1 unsubtyped (*n* = 17), mixed infection (*n* = 17), or indeterminate (*n* = 9) with the Abbott RealTime HCV Genotype II assay. Genotype 1 was confirmed in 14/17 samples (82%): eight resulted in subtype 1b, and five resulted in subtype 1a with both DS and UDPS, while one was classified as subtype 1e by DS and mixed infection (1e + 1a) by UDPS. Three of seventeen genotype 1 samples resulted in genotype 3h with both sequencing approaches. Only one mixed infection was confirmed by UDPS (4d + 1a), while in 88% of cases a single component of the mixture was detected (five genotype 1a, four genotype 1b, two genotype 3a, two genotype 4m, and two genotype 4d); 44% of indeterminate samples resulted genotype 2c by both DS and UDPS, 22% resulted genotype 3a; one indeterminate sample by Abbott resulted in genotype 4d, one resulted in genotype 6n, and one was classified as subtype 3a by DS, and resulted mixed infection (3a + 3h) by UDPS. The concordance between DS and UDPS was 94%, 88%, and 89% for genotype 1, co-infection, and indeterminate results, respectively. UDPS should be considered very useful to resolve ambiguous HCV genotyping results.

## 1. Introduction

Hepatitis C virus (HCV) is the unique recognized member of the genus *Hepacivirus* in the *Flaviviridae* family, including positive-strand RNA viruses. Based on phylogenetic analysis, HCV has been classified into seven different genotypes and 67 confirmed subtypes; divergence of the whole genome sequence is over 30% for genotypes and between 15% and 30% for subtypes [1]. Although sequencing of conserved HCV genome regions (core/E1 or the NS5B) is the reference method for assigning HCV genotype/subtype [2], several molecular methods have been established for HCV genotyping in diagnostic routine. The most used commercial assays in clinical practice are Versant HCV Genotype 2.0 assay and the Abbott RealTime HCV Genotype v 2.0. Both assays use the 5′ untranslated region (5′ UTR) to define HCV 1–6 genotypes [3], and additional targets to define the subtype. In fact, while HCV subtyping is not clinically relevant for Peg-interferon-α (PegIFN-α) and Ribavirin (RBV) treatment regimens, it is considered relevant in the era of directly-acting antivirals (DAAs), because of considerable HCV genotype and/or subtype-driven differences in response rates and resistance patterns [4,5,6]. The additional HCV genome targets used for subtyping are in the core region for the Versant HCV Genotype 2.0 and in the NS5B region for the Abbott RealTime HCV Genotype v 2.0. Furthermore, Abbott improved the performance of RealTime HCV Genotype II (targeting the 5′ UTR and NS5B regions), with the ABBOTT Genotype PLUS RUO test, where the core region is used to further characterize genotype 1 unsubtyped samples [7]. Despite the technical improvements in HCV genotyping tools, indeterminate, mixed and unspecified subtype results are obtained in a small but not irrelevant proportion of samples in the daily clinical practice [8]. In addition, in some cases misclassification of genotype/subtype has been provided by commercial assays. Hence, at present, the correct genotyping/subtyping of these samples remains a big challenge in the perspective of proper therapeutic regimen assignment.

Sequencing of a conserved HCV genome region (i.e., NS5B) by direct approach is considered the reference method, but due to the low sensitivity towards minority variants present in the viral population, it is unable to highlight the presence of mixed infections [9,10]; to this respect, ultra-deep pyrosequencing (UDPS) with next generation sequencing platforms is the method of choice, due to the massive output of clonal sequences data that allows an in depth analysis of complex viral populations present in each single sample to be performed [11]. In this study, we used sequencing of NS5B by direct sequencing (DS) and UDPS methods to re-evaluate 43 samples that resulted indeterminate, mixed infection or not subtyped genotype 1 with the Abbott RealTime HCV Genotype II assay.

## 2. Results

The NS5B amplicons obtained from 43 plasma samples (genotype 1, *n* = 17; indeterminate HCV genotype, *n* = 9; HCV multiple infections, *n* = 17; median of HCVRNA viral load (IU/mL): 6.26 × 10^5^; (interquartile range, IQR): 1.04 × 10^5^ − 3.11 × 10^6^) and successfully amplified with the protocol described by Quer et al. [8] were analyzed by DS and UDPS. The results compared to those obtained with the Abbott RealTime HCV Genotype II assay are shown in Table 1.

**Figure 1:**
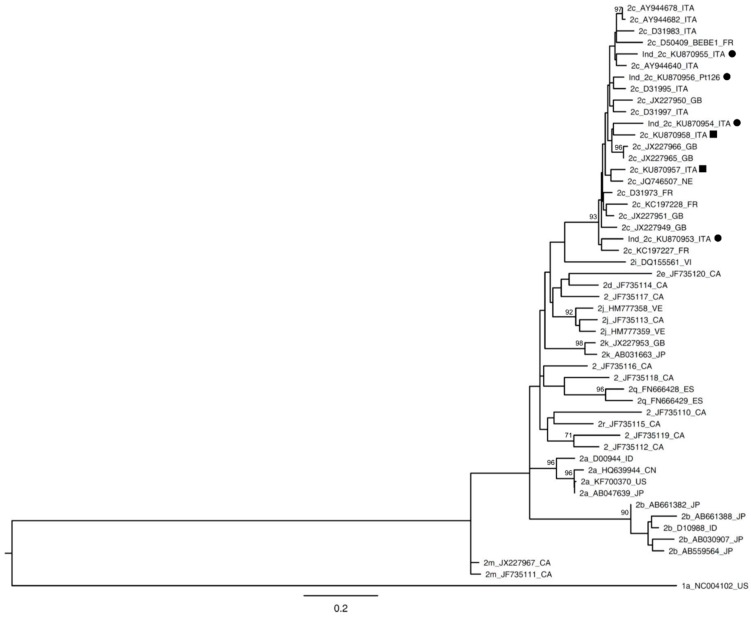
NS5B phylogenetic tree of genotype 2c samples. Bootstrap analysis with 500 replicates is performed to assess the significance of the nodes; values greater than 70% are considered significant and reported in the figure. A genotype 1a sequence (1a_NC004102_US) is used as the outgroup. Sequences from patients with an indeterminate result by Abbott RealTime HCV Genotype II assay are indicated with a circle (●). Reference sequences (1a_NC004102_US; 2c_D50409_BEBE 1_FR) are available in the Los Alamos National Library HCV sequences database [12] and were previously reported as reference sequences by Smith et al. [1]. Sequences indicated with squares (■) represent the 2c sequences correctly genotyped as genotype 2 by Abbott assay. The bar represents substitution per nucleotide position. CA, Canada; CN, China; DK, Denmark; ES, Spain; FR, France; GB, Great Britain; ID, Indonesia; ITA, Italy; JP, Japan; NE, Nederland; US, United States; VE, Venezuela; VI, Vietnam.

Sequencing analysis confirmed the presence of genotype 1 in 14 samples (14/17, 82%). Among genotype 1 samples, eight resulted in subtype 1b, five resulted in subtype 1a when analyzed with both methods, DS and UDPS; while one sample, classified as subtype 1e by DS, was found to be a mixed infection of HCV subtype 1e (2320 reads, 98.85%) and 1a (27 reads, 1.15%) by UDPS. Interestingly, three genotype 1 samples resulted to be genotype 3 subtype h with both methods. Among the samples classified as HCV mixed infection by Abbott RealTime HCV Genotype II commercial assay, all samples returned a monoinfected with the Sanger method, whereas one sample was confirmed as a mixed infection by UDPS (Pt 89–114b). Nevertheless, this discrepancy was expected since, in all samples, the subtype detected only by UDPS represented <10% of the total viral population. Among indeterminate samples by the Abbott assay, 44% of specimens resulted in the 2c genotype. All 2c samples belonged to Italian patients. Since this was the first occurrence of a large number of indeterminate genotypes reclassified as 2c, we carried out a phylogenetic analysis of these 2c samples using NS5B sequences obtained by DS. As shown in Figure 1, the 2c samples did not fall into a specific cluster and they resulted in being interspersed among other samples, both from Italy and from other European countries. Two 2c sequences from samples correctly identified as genotype 2 by the Abbott assay were included in this phylogenetic analysis, and they showed a mean identity of 91.35% (range: 90.33–92.49) with the 2c sequences previously identified as “indeterminate” by the Abbott assay.

**Figure 2:**
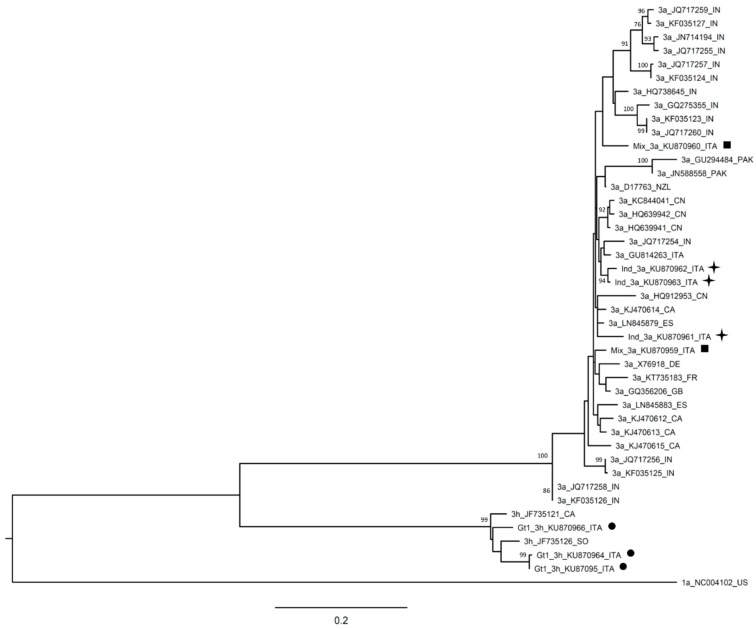
NS5B phylogenetic tree of genotype 3 samples. Bootstrap analysis with 500 replicates is performed to assess the significance of the nodes; values greater than 70% are considered significant and reported in the figure. A genotype 1a sequence (1a_NC004102_US) is used as the outgroup. Sequences from patients resulted genotype 1, mixed infection or indeterminate by Abbott RealTime HCV Genotype II assay are indicated with a circle (●), a square (■), or a star (✦), respectively. Reference sequences (3a_D17763; 3h_JF735121) are available in the Los Alamos National Library HCV sequences database [12] and were previously reported as reference sequences by Smith et al. [1]. The bar represents substitution per nucleotide position. CA, Canada; CN, China; DE, Germany; FR, France; GB, Great Britain; IN, India; ITA, Italy; NZ, New Zeland; PK, Pakistan; SO, Somalia.

Among samples classified as indeterminate using the Abbott assay, 3/9 (33.3%) resulted in genotype 3a using DS. Similar to genotype 2c, genotype 3a samples were also interspersed among other samples both from Italy and from other countries (Figure 2).

Moreover, the similarity in GenBank with the other 2c (GenBank accession number: KU870953-KU870958), 3a (GenBank accession number: KU870959-KU870963), and 3h (GenBank accession number: KU870964-KU870966) sequences revealed that they did not represent new variants.

The concordance rate between the two methods is shown in Table 2.

Interestingly, Pt 76 (co-infected genotype 3 + 4 with Abbott assay) seems to be mono-infected (genotype 3 subtype “a”) if analysed with UDPS, considering the threshold of 1%. However, a second haplotype (genotype 4 subtype “d”) is present with 24 reads.

## 3. Discussion

Several authors have shown that the methods for HCV genotype determination could give erroneous results [8,13,14] or that they could exhibit discrepancies among the results obtained with different methods. The Sanger method for the NS5B region is considered the gold standard for proper HCV genotyping. However, over the past few years, to increase the accuracy the UDPS approach has been proposed as a Sanger alternative [15]. We have previously shown that UDPS accurately identifies the HCV genotype in samples clearly determined by the Abbott assay [16,17,18]. In this study, we wanted to assess the capability of UDPS in comparison to DS in the correct classification of samples, which had no conclusive result with the Abbott RealTime HCV Genotype II assay.

Concerning the subtyping of genotype 1 samples, the concordance rate between DS and UDPS was 94% (16/17). In only one sample (Pt 133), the UDPS showed the presence of a second subtype (1a, reads 27/2347; 1.15%) that could not be evidenced with the Sanger method. With regard to indeterminate genotypes, 44% of them resulted to be genotype 2c. The phylogenetic analysis, which also included 2c genotypes properly genotyped as 2 by the Abbott commercial assay, did not reveal the presence of particular variants (Figure 1), suggesting that the low performance of the Abbott RealTime HCV Genotype II commercial assay with the 2c subtype was not related to the particular viral sequences harbored in the samples, but to a more general method limitation, possibly related to probe inadequacy.

Notably, both samples with HCV mixed infection including genotype 2 (1b + 2, *n* = 2) were not confirmed by UDPS, suggesting a probable cross-reaction of genotype 2 probes with genotype 1.

Troubles in the correct identification of genotype 2 had already been described by other authors [14]. In fact, Vaghefi et al. [14] described a case of 2f genotype being classified as 5 by the Abbott assay. Furthermore, our data confirm the results reported by Gonzalez et al. [19] on the weak ability of commercial assay to properly detect genotype 3 subtype a (Pt 68, Pt 102, and Pt 103).

The existence of coinfections has been described by several authors, but their frequency varies in accordance to the method in use [8,9,10,11,12,13,14,15,16,17,18,19,20] and the considered population [21,22]. The Abbott test showed the presence of 2.23% mixed infections in our routine activity; 17 of them were analyzed both with DS and UDPS. Only one sample was confirmed as “mixed infection” (1a + 4d) by UDPS, while DS gave only the genotype 4d as a result. However, this does not represent a discrepancy, because the Sanger method can identify sequences representing at least 20% of the total population. In UDPS analysis, the 1a sequences represented only the 10% (81/806 reads) of the total reads, thus, this proportion could not be detected by DS. It is to be pointed out that failure of UDPS to identify mixed infections may be due to reduced sensitivity for minority variants as compared to the Abbot assay. In fact, for UDPS, we defined mixed infection as those where the presence of more than one haplotype was detected with a frequency of at least 1%. In this respect, we have adopted the same cut off adopted by Quer et al. [8].

It is worth noting that Quer et al. [8] observed cross-reactivity between genotype 4f and 5 in the Abbott assay. In fact, all Spanish samples classified as mixed infection (4 + 5) resulted genotype 4f if analysed with UDPS or DS. Unlike the results described by Quer et al. [8], in our specimens Abbott assay never gave 4 + 5 co-infection. This is probably due to cross-reactivity between genotype 5 with subtype 4f and not with subtype 4d, the main subtype observed in Italy.

Although a limited number of samples were analyzed in our study, some considerations can be drawn. First, in considering of the high genotype and subtype specificity of the activity of new drugs DAA [23], genotype 1 samples without subtype assignment by commercial assays need re-evaluation based on sequencing. In this respect, our results confirm previous observations [8] and add novel evidence in this field. In particular, differently from previous studies, as many as 3/19 (16%) genotype 1 samples were re-classified as genotype 3h by both DS and UDPS. This may be clinically relevant, as the different sensitivity to drugs of genotype 3 in comparison to genotype 1 and inaccurate subtyping may lead to an incorrect choice of antiviral therapy, resulting in treatment failure [24]. Genotypes 1 and 3 are treated with different new DAA regimens [25,26], therefore, misclassification may have a detrimental effect on the therapeutic success rate. However, in our routine practice, samples with ambiguous results, or classified as genotype 1 (unsubtyped) by the Abbott assay, are retested by Sanger sequencing to establish the precise genotype/subtype, and the therapeutic decision is taken according to the sequencing results. Hence, the initial misclassification does not have a negative impact on therapy regimen choice. In fact, we have no evidence of therapeutic failures in these patients.

The same considerations apply to samples resulting indeterminate genotype by the Abbott assay; in fact, 4/9 (44%) of indeterminate samples were classified as genotype 2c by both DS and UDPS. Second, the samples resulting mixed infection with commercial assays need re-evaluation, since as many as 94% of them resulted as single infection by UDPS. Furthermore, DS does not appear to be the method of choice for mixed infection due to the low sensitivity for minority variants. To date, UDPS have limited use because of results elaboration complexity, as well as the need of higher expertise, to determine the error rate threshold. Moreover the facility and costs are complex when performing in a real-life clinical setting. However, the development of methods to assess the actual presence of minority variants [11,27] and the recent availability commercial kits, such as SENTOSA SQ HCV genotyping assay (VELA DIAGNOSTICS Germany GMBH, Hamburg, Germany) based on next-generation sequencing, may encourage the use of the UDPS technique routinely in HCV genotyping.

## 4. Materials and Methods

### 4.1. Patients

Between November 2011 and September 2015, a total of 3179 HCV genotyping tests were performed in our laboratory on plasma samples using an Abbott RealTime HCV Genotype II assay (Abbott Laboratories, Des Plaines, IL, USA), according to the manufacturer’s instruction. Among these, 208 samples (6.54%) had inconclusive/equivocal or insufficiently precise results. In particular, 71 (2.23%) were classified as co-infections, 28 (0.88%) were indeterminate, and 109 (3.43%) were genotype 1 unsubtyped. Within the 208 samples, 109 plasma (genotype 1 unsubtyped, *n* = 52; mixed infection, *n* = 46; indeterminate genotype, *n* = 11) were available for retrospective retesting and, thus, were included in our study. A total of 47 samples (43%) were successfully amplified with the protocol described by Quer et al. [8]. Of these, 43 samples (genotype 1, *n* = 17; indeterminate HCV genotype, *n* = 9; HCV multiple infections, *n* = 17) were retested by DS in NS5B region and the same amplicons were analyzed by the UDPS approach.

As a quality control to the methods, four samples with unequivocal results (two with genotype 1b, one genotype 4, and one genotype 5), were sequenced with both approaches, confirming the results obtained with the Abbott test [28].

### 4.2. Ethical Issues

The specimens represented residual samples from routine laboratory activity, and were anonymized before the inclusion in the study. Approval for the use of anonymized residual samples for research investigation was obtained by the local ethics committee (Ethics Committee of INMI “Spallanzani”) (Statement n° 49/2013).

### 4.3. HCV Genotyping by Commercial Assay

Abbott RealTime HCV Genotype II (Abbott Laboratories) was used in the diagnostic routine activity to determine the HCV genotype based on dual-target real-time PCR: the 5′ UTR region represented the target to discriminate between HCV genotypes, and the *NS5B* gene was the target for 1a and 1b subtyping.

### 4.4. RNA Extraction

HCV RNA was extracted from 400 μL plasma or serum samples by QIAsymphony DSP Virus/Pathogen Midi Kit (Qiagen GmbH, Hilden, Germany) using the automated QIAsymphony instrument (Qiagen).

### 4.5. RT-PCR Amplification for DS and UDPS

The process of RT-PCR amplification for DS and UDPS was performed using a heminested PCR protocol previously described by Quer et al. [8]. A final product of 454 nucleotides (targeting NS5B region) was obtained.

### 4.6. Direct Sequencing (DS) and Ultra-Deep Pyrosequencing (UDPS)

Sequencing was performed on the automated ABI Prism 3100 instrument, by using a BigDye Terminator cycle sequencing kit (Applied Biosystems, Warrington, UK).

UDPS was performed with GS Junior 454 (Roche Diagnostics GmbH, Mannheim, Germany) according to manufacturer’s instructions, following the protocol described by Quer et al. [8]. Multiplex identifiers (MIDs) for sample barcoding, as well as adaptors for UDPS, were added to the sequence primers in the second PCR round.

A mean of 4458 (range: 309–9829) reads for each sample was obtained. All reads shorter than 400 bp, with more than one mismatch on the MID, two on the specific primer, three on the universal primer M13, and with indels, or showing more than three gaps were discarded. The primers and M13 sequences were trimmed and reverse sequences were reverted. All identical sequences were clustered using CD-HIT software (http://www.bioinformatics.org/cd-hit/) [29]. Haplotype sequences were identified as the most frequent representative reads and their frequencies computed as the number of observed reads identical to 90%. For the definition of mixed infection with UDPS, we established a threshold of a minimum of five reads and 1% of the reads’ abundance in the single sample, according to a previous study [8]. For each haplotypes, the genotype was identified comparing the similarity with the reference sequences of the genotype reported in Smith et al. [1].

### 4.7. Phylogenetic Analysis

The nucleotides’ Sanger sequences of patients with the 2c genotype and 3 genotype were analysed separately. In both cases the sequences were aligned, using the Muscle program v3.8.31 [30], with the most representative sequences of genotype 2, available in GenBank. The best fit model was identified (considering the Bayesian Information Criterion (BIC) and the Akaike Information Criterion (AIC)) and a phylogenetic analysis was performed using the maximum-likelihood method with the Kimura two-parameter model + G, implemented in MEGA6 software [12]. To evaluate the robustness, the bootstrap probabilities were estimated with 500 replications.

### 4.8. Nucleotide Sequence Accession Numbers

The Sanger sequences obtained in this study have been deposited in GenBank under accession numbers KU870927-KU870937, KU870939, KU870941-KU870966, KU870968-KU870973, KU870975.

## 5. Conclusions

In conclusion, at present, accurate HCV genotyping/subtyping is mandatory to select the most appropriate DAA and to reduce the risk of therapy failure. Our findings confirm that commercial assays, based on the RealTime method, may be inaccurate in some particular situations that require methods with higher resolution. In this respect, DS and UDPS may be of relevant help: sequencing-based subtyping may resolve genotype 1 and equivocal genotype samples; in this case UDPS does not offer additional advantages as compared to DS. However UDPS allows accurate identification of HCV multiple infections that are not appreciated by DS, and is able to rule out inaccurate assignment of mixed infections by commercial methods. Thus, the higher resolution power of UDPS allows both the identification of all haplotypes present in the sample (also, the minor variants of the viral population), and the clarification of the ambiguous results.

## Figures and Tables

**Table 1:** Comparison of genotyping results obtained by Abbott RealTime HCV Genotype II commercial assay, direct sequencing (DS) and ultra-deep pyrosequencing (UDPS). Four patients (patient, Pt 49–59, Pt 16–50, Pt 89–114, and Pt 54–127) had two samples collected on different dates, indicated with “a” and “b”. The indeterminate result obtained by the Abbott assay is indicated with “ind”. For Pt 55 no results (NR) have been obtained with UDPS.

Patient Code	HCV RNA	Date of Collection	Abbott RealTime HCV Genotype II	DS	UDPS
Pt 12	2.25 × 10^6^	17 May 2013	1	1b	1b
Pt 108	5.63 × 10^3^	26 February 2015	1	1b	1b
Pt 29	1.58 × 10^4^	02 November 2013	1	1b	1b
Pt 10	1.50 × 10^5^	13 May 2013	1	1b	1b
Pt 17	6.87 × 10^4^	06 July 2013	1	1b	1b
Pt 22	1.88 × 10^5^	31 August 2013	1	1b	1b
Pt 141	5.71 × 10^3^	7 August 2015	1	1b	1b
Pt 142	4.49 × 10^4^	7 August 2015	1	1b	1b
Pt 53	1.70 × 10^6^	29 April 2014	1	1a	1a
Pt 8	7.06 × 10^6^	15 February 2013	1	1a	1a
Pt 13	2.31 × 10^5^	3 June 2013	1	1a	1a
Pt 14	9.12 × 10^6^	12 June 2013	1	1a	1a
Pt 23	7.90 × 10^6^	13 September 2013	1	1a	1a
Pt 133	2.63 × 10^6^	10 June 2015	1	1e	1e + 1a
Pt 49–59a	7.72 × 10^5^	31 March 2014	1	3h	3h
Pt 49–59b	4.53 × 10^5^	20 June 2014	1	3h	3h
Pt 77	6.77 × 10^6^	15 September 2014	1	3h	3h
Pt 54–127a	4.94 × 10^6^	2 May 2014	1a + 3	1a	1a
Pt 54–127b	2.68 × 10^6^	12 June 2015	1a + 3	1a	1a
Pt 81	6.27 × 10^6^	29 September 2014	1a + 3	1a	1a
Pt 44	3.93 × 10^6^	25 February 2014	1a + 4	1a	1a
Pt 128	5.25 × 10^5^	17 June 2015	1a + 4	1a	1a
Pt 55	6.49 × 10^6^	15 May 2014	1a + 4	1a	NR
Pt 92	1.96 × 10^5^	4 December 2014	1b + 3	1b	1b
Pt 18	3.72 × 10^4^	5 August 2013	1b + 2	1b	1b
Pt 97	1.31 × 10^5^	19 December 2014	1b + 2	1b	1b
Pt 115	5.32 × 10^5^	17 March 2015	1b + 4	1b	1b
Pt 76	2.83 × 10^6^	12 September 2014	3 + 4	3a	3a
Pt 46	1.04 × 10^5^	20 March 2014	3 + 4	3a	3a
Pt 16–50a	6.26 × 10^5^	2 July 2013	1 + 4	4m	4m
Pt 16–50b	1.21 × 10^6^	1 April 2014	1 + 4	4m	4m
Pt 104	1.14 × 10^6^	4 February 2015	1a + 4	4d	4d
Pt 89–114a	6.10 × 10^6^	24 October 2014	1 + 4	4d	4d
Pt 89–114b	2.77 × 10^6^	10 March 2015	1a + 4	4d	4d + 1a
Pt 41	2.15 × 10^6^	15 January 2014	ind	2c	2c
Pt 84	7.57 × 10^4^	2 October 2014	ind	2c	2c
Pt 126	1.83 × 10^5^	24 April 2015	ind	2c	2c
Pt 123	3.11 × 10^6^	9 May 2015	ind	2c	2c
Pt 102	8.92 × 10^4^	2 February 2015	ind	3a	3a
Pt 103	3.97 × 10^6^	2 February 2015	ind	3a	3a
Pt 56	6.17 × 10^3^	24 May 2014	ind	4d	4d
Pt 94	1.01 × 10^4^	10 December 2014	ind	6n	6n
Pt 68	2.53 × 10^5^	28 July 2014	ind	3a	3a + 3h

**Table 2:** Concordance between direct sequencing (DS) and ultra-deep pyrosequencing (UDPS) in the HCV genotype analysis.

Genotype Identified by Abbott	Number of Samples Tested by Abbott	Number of Samples Identified by DS (Type of Infection)	Number of Samples Identified by UDPS	Concordance (UDPS vs. DS)
1	17	17 (mono-infection)	16 mono-infection	94%
			1 co-infection: 1e + 1a	
co-infection	17	17 (mono-infection)	15 mono-infection	88%
			1 co-infection: 4d + 1a	
Indeterminate	9	9 (mono-infection)	8 mono-infection	89%
			1 co-infection: 3a + 3h	
Total	43	43	42	
